# LncRNA MAFG-AS1 regulates miR-125b-5p/SphK1 axis to promote the proliferation, migration, and invasion of bladder cancer cells

**DOI:** 10.1007/s13577-020-00470-3

**Published:** 2021-01-05

**Authors:** Chenye Tang, Yuntao Wu, Xiao Wang, Kean Chen, Zhiling Tang, Xiao Guo

**Affiliations:** Department of Urology, Jiaxing Second Hospital, No.1518 North Ring Road, Jiaxing City, 314000 Zhejiang Province People’s Republic of China

**Keywords:** Bladder cancer, MAFG-AS1, miR-125b-5p, SphK1

## Abstract

MAFG-AS1 is an oncogenic lncRNA in multiple types of cancer. However, its role in bladder cancer (BC) remains unclear. The present study aimed to investigate the function of MAFG-AS1 in BC. BC and paired non-tumor tissues were collected. Two BC cell lines HT01197 and HT-1376 were used. Dual luciferase activity assay, RT-qPCR, western blot, CCK-8, transwell invasion assay, and wound healing assay were performed. We found that MAFG-AS1 was significantly up-regulated in BC tissues and predicted a poor survival rate. MAFG-AS1 interacted with miR-125b-5p. However, the expression levels of MAFG‑AS1 and miR-125b-5p were not obviously correlated in BC tissues, and MAFG‑AS1 and miR-125b-5p did not regulate the expression of each other. Interestingly, we found that SphK1, a downstream target of miR-125b-5p, was negatively correlated with miR-125b-5p, while it was positively correlated with MAFG-AS1 across BC tissues. In addition, overexpression of MAFG‑AS1 upregulated the expression of SphK1 in BC cells, and attenuated the inhibitory effects of miR-125b-5p on the expression of SphK1. Functional assays showed that overexpression of MAFG‑AS1 promoted BC cell proliferation, migration, and invasion, while its effects were attenuated by overexpression of miR-125b-5p. Moreover, overexpression of miR-125b-5p inhibited BC cell proliferation, migration, and invasion, while its effects were alleviated by overexpression of SphK1. Taken together, our findings demonstrated that MAFG-AS1 has an oncogenic role in BC by regulating the miR-125b-5p/SphK1 axis. MAFG-AS1 might serve as a good diagnostic marker and a potential therapeutic target of BC.

## Introduction

Bladder cancer (BC) affected 549,393 new cases, accounting for 3.0% of all new cancer cases, and caused 199,922 deaths, accounting for 2.1% of all cancer deaths in 2018 worldwide [1]. The high mortality rate of BC is mainly caused by the existence of tumor metastasis in a considerable portion of BC patients by the time of initial diagnosis [2]. However, effective therapies for advanced BC remain lacking, leading to poor prognosis [3]. The main risk factor of BC is the exposure to carcinogens, such as tobacco, which can induce BC by hypermethylation and DNA damage [4, 5]. However, exposure to carcinogens is not sufficient for the occurrence and development of BC. In effect, genetic alterations, such as germline mutations or epigenetic modification, independent from carcinogen, contribute to the occurrence of BC [6].

Characterization of the molecular pathogenesis of BC provides potential therapeutic targets for BC [7]. Sphingosine kinase 1 (SphK1) can phosphorylate sphingosine to form sphingosine-1-phosphate (S1P), which plays critical roles in the regulation of cancer cell proliferation and survival in different types of cancer [8, 9]. Inhibition of SphK1 is considered as a potential target for the treatment of BC [10]. For instance, tumor suppressive miRNA miR-125b-5p can target SphK1 to inhibit invasion and migration of bladder cancer cells [11]. Long non-coding RNA (lncRNA) MAFG-AS1 has been characterized as an oncogenic lncRNA in several types of cancer, such as colorectal cancer and hepatocellular carcinoma [12, 13]. For instance, MAFG-AS1 can sponge miR-147b to activate NDUFA4, thereby promoting the proliferation of colorectal cancer cells [12]. MAFG‑AS1 can downregulate miR‑6852 to promote hepatocellular carcinoma cell invasion, migration and proliferation [13]. Therefore, MAFG‑AS1 participates in cancer biology mainly by interacting with microRNAs (miRNAs). However, its involvement in BC is unknown. We analyzed The Cancer Genome Atlas (TCGA) and Genotype-Tissue Expression (GTEx) datasets and observed the upregulation of MAFG-AS1 in BC. In addition, we predicted that MAFG-AS1 could interact with miR-125b-5p to participate in BC. This study was, therefore, performed to investigate the possible interaction between MAFG-AS1 and miR-125b-5p in BC.

## Materials and methods

### Research patients

This study enrolled 66 patients with BC (42 males and 24 females, age range 40–69 years, mean age 54.2 ± 6.2 years) admitted at Jiaxing Second Hospital between June 2012 and June 2014. This study was approved by the Ethics Committee of Jiaxing Second Hospital (approval number: 2012-JSH-07). The BC patients were all newly diagnosed cases. No previous history or family history of malignancies was observed. No anti-cancer therapy was performed before admission. Patients diagnosed with multiple clinical disorders were excluded. Bladder biopsy was performed on all patients to collect BC and paired non-tumor tissues (urothelial tissues within 3 cm around tumors). All tissues were confirmed by histopathological exam. All patients and their families signed the informed consent before enrollment. Tissue specimens were stored in a − 80 °C freezer before the subsequent experiments.

### Treatment and follow-up

The 66 patients included 5, 15, 23 and 23 cases at AJCC clinical stage I, II, III, and IV, respectively. All patients received partial or radical cystectomy. A 5-year follow-up was performed on all patients from the date of admission. All patients completed the follow-up study. Survival conditions were recorded and used to plot survival curves.

### BC cells and transient transfection

BC cell lines HT-1197 and HT-1376 (ATCC, USA) were used. Cell culture medium was composed of 90% DMEM medium and 10% FBS (Invitrogen, Shanghai, China). Cells were cultivated in a 5% CO_2_ incubator at 37 °C with 95% humidity to reach about 85% confluence. MiR-125b-5p mimic (5′-UCCCUGAGACCCUAACUUGUGA-3′) and negative control (NC) miRNA (5′-AGUGUGGGGCAUGUAGUCAACC-3′) were obtained from Sigma-Aldrich (USA). PcDNA3.1 vector was used to construct MAFG-AS1 (NCBI Accession: NR_015454.1) and SphK1 (NCBI Accession: NR_015454.1) expression vectors. HT-1197 cells were transfected with 10 nM vector or 45 nM miRNA using lipofectamine 2000 (Invitrogen, USA). To perform NC experiments, cells were transfected with empty pcDNA3.1 vector or NC miRNA. The untransfected cells were used as the Control (C). Cells were harvested at 48 h to perform the subsequent experiments.

### Dual luciferase activity assay

The pGL3 vector (Promega Corporation) was used to construct MAFG-AS1 vector. Lipofectamine 2000 (Catalog 11668019, Invitrogen) was used to co-transfect MAFG-AS1 vector + miR-125b-5p (miR-125b-5p group) or MAFG-AS1 vector + NC miRNA (NC miRNA group) into HT-1197 cells. Dual Luciferase Assay System (Catalog #60683-1, BPS Bioscience) was used to measure luciferase activity at 48 h post-transfection.

### RNA preparation

Total RNAs were extracted from BC and non-tumor tissues as well as HT-1197 cells using Trizol reagent (Catalog 15596026, Invitrogen). To harvest miRNAs, 85% ethanol was used to precipitate and wash RNA samples. All RNA samples were digested with gDNA eraser (Catalog RR047A, Takara) to remove genomic DNAs.

### RT-qPCR assay

PrimeScript RT Reagent Kit (Catalog RR047A, Takara) was used to reverse transcribe total RNAs into cDNA. SYBR Green RT-PCR Kit (Catlog Q111, Vazyme Biotech) was used to prepare qPCR reactions. The expression levels of MAFG-AS1 and SphK1 were determined with GAPDH as an endogenous control. Measurement of the expression levels of mature miR-125b-5p was performed using All-in-One™ miRNA qRT-PCR Detection Kit (Catalog QP015, Genecopoeia) with all steps completed following the manufacturer’s instructions. The Ct values of 3 replicate reactions were processed using Ct (ΔΔCt) method to calculate fold changes of gene expression levels across samples. Primer sequences were: 5′-CGAAGATCTCCTCACCTCCC-3′ (forward) and 5′-TTAAAGCCGGTCGTGGAGAT-3′ (reverse) for MAFG-AS1; 5′-AGCTTCCTTGAACCATTATGCTG-3′ (forward) and 5′-AGGTCTTCATTGGTGACCTGCT-3′ (reverse) forSphK1; 5′-GGTGATTGTGGTGAGCGTGTT-3′ (forward) and 5′-AGGCCACATCAATGAGGAAGA-3′ (reverse) for GAPDH. Forward primer of miR-125b-5p was 5′-TCCCTGAGACCCTAACTTG-3′. Universal reverse primer and U6 forward primer were from the kit.

### Western blot assay

Total proteins were extracted from transfected cells using RIPA solution (Catalog 89901, Invitrogen). Protein concentrations were measured using BCA method (Catalog P0011, Beyotime). Denaturation of protein samples was performed in boiling water for 15 min. PVDF membrane was used to transfer proteins, and PBS containing 5% non-fat milk was used to block the membranes. SphK1 (ab71700, Abcam) and GAPDH (ab9485, Abcam) rabbit primary antibodies were used to incubate with membranes at 4 °C for 16 h, followed by incubation with HRP Goat Anti-Rabbit (IgG) secondary antibody (ab97051, Abcam) at room temperature for 2 h. Signals were produced using ECL (Catalog GERPN2105, Sigma-Aldrich) and processed using Quantity One software.

### CCK-8 assay

Cell counting Kit-8 kit (CCK-8, Catalog MBS176412, MyBioSource) was used to perform cell proliferation assay in HT-1197 cells harvested at 48 h post-transfection. In brief, 4000 cells in 0.1 ml medium were transferred to each well of a 96-well plate. Cells were cultivated at 37 °C and OD values at 450 nm were measured every 24 h for a total of 96 h. CCK-8 solution was added into each well at 2 h before the measurement of OD values.

### Transwell invasion assay

Transwell invasion assay was performed to detect the invasion ability of HT-1197 cells. In brief, transwell inserts were first treated with 50 μl matrigel (BD Biosciences, USA) at 37 °C for 6 h. Afterwards, 10^5^ HT-1197 cells were seeded into the upper chamber of matrigel-coated transwell inserts, and 200 ml DMEM containing 20% FBS was added into the lower chamber. After incubation for 24 h, the noninvasive cells were removed with a cotton swab. The invasive cells were stained with 0.1% crystal violet for 20 min, and then were counted using an optical microscope.

### Wound healing assay

Wound healing assay was performed to assess the migration ability of HT-1197 cells. A total of 10^6^ HT-1197 cells were seeded into a 6-well plate. After reaching 80% confluency, the cell monolayers of HT-1197 cells were scratched by a pipette tip to generate the wound. The debris and floating cells were cleaned by washing with PBS. The photographic images were taken at 0 h and 24 h after scratch.

### Statistical analysis

All experiments were performed in 3 independent biological replicates. Data were expressed as the mean ± SD values. The average values were used to express gene expression levels in paired tissues. Unpaired *t* test was used to compare relative luciferase activities. Paired *t* test was used to compare gene expression levels between BC and non-tumor tissues. Comparisons among multiple groups were performed using ANOVA (one-way) combined with Tukey test. Survival curves were plotted for high and low MAFG-AS1 level groups (*n* = 32, the median level of MAFG-AS1 in BC tissues as cutoff value) and were compared by log-rank test. Correlations between the expression levels of MAFG-AS1 and patients’ clinical data were analyzed by Chi-squared test. Correlations between MAFG-AS1 and miR-125b-5p, between miR-125b-5p andSphK1, and between MAFG-AS1 and SphK1 were analyzed by Pearson’s correlation coefficient. *p* < 0.05 was considered as statistically significant.

## Results

### MAFG-AS1 interacted with miR-125b-5p

**Fig. 1:**
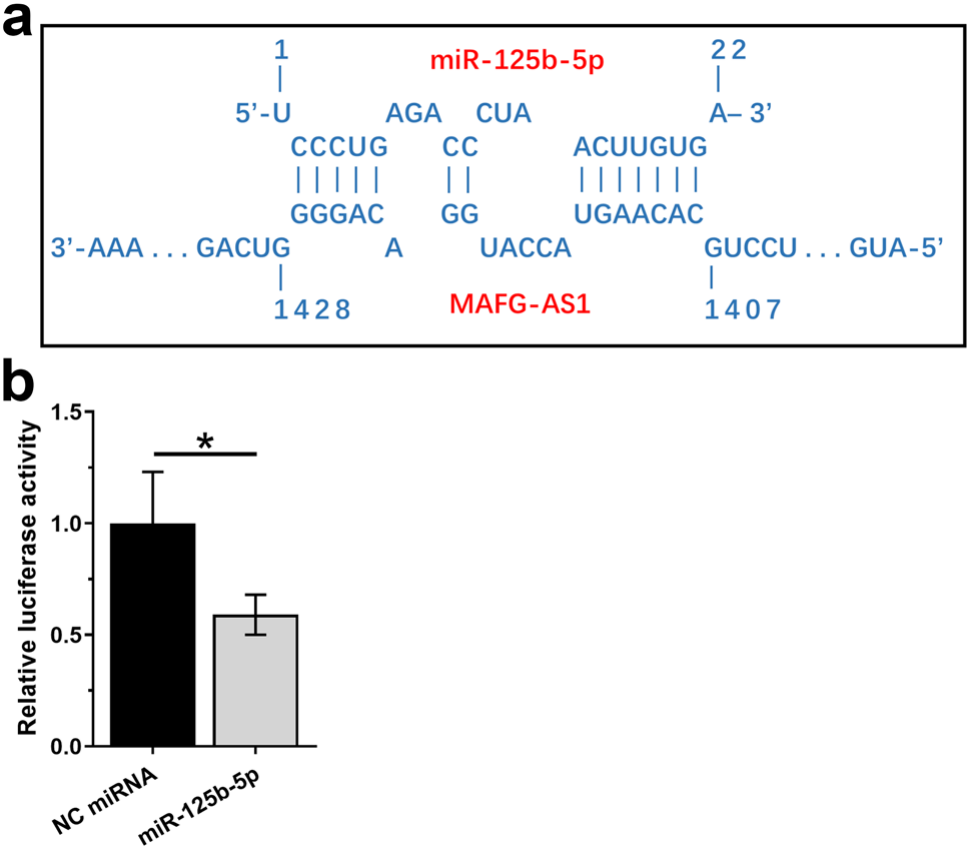
MAFG-AS1 interacted with miR-125b-5p. Possible base pairs formed by MAFG-AS1 and miR-125b-5p were predicted using IntaRNA (**a**). Dual luciferase activity assay was performed by co-transfecting MAFG-AS1 vector + miR-125b-5p (miR-125b-5p group) or MAFG-AS1 vector + NC miRNA (NC miRNA group) into HT-1197 cells. Luciferase activity was measured at 48 h post-transfection. This experiment was repeated 3 times and mean values were presented (**b**). *, *p* < 0.05

Possible base pairing between MAFG-AS1 and miR-125b-5p was predicted using IntaRNA 2.0 software [14]. It was observed that MAFG-AS1 and miR-125b-5p may form strong base pairing (Fig. 1a). Dual luciferase activity assay was performed by co-transfecting MAFG-AS1 vector + miR-125b-5p (miR-125b-5p group) or MAFG-AS1 vector + NC miRNA (NC miRNA group) into HT-1197 cells. Compared to NC miRNA group, the relative luciferase activity was significantly lower in miR-125b-5p group (Fig. 1b, *p* < 0.05). In the luciferase vector of MAFG-AS1, sequence of luciferase gene is at the downstream of MAFG-AS1. Therefore, miR-125b-5p may bind to the RNA transcript composed of MAFG-AS1 and luciferase gene mRNA, thereby suppressing the translation of the luciferase gene and reducing luciferase activity.

### MAFG-AS1 was upregulated in BC and predicted poor survival

**Fig. 2:**
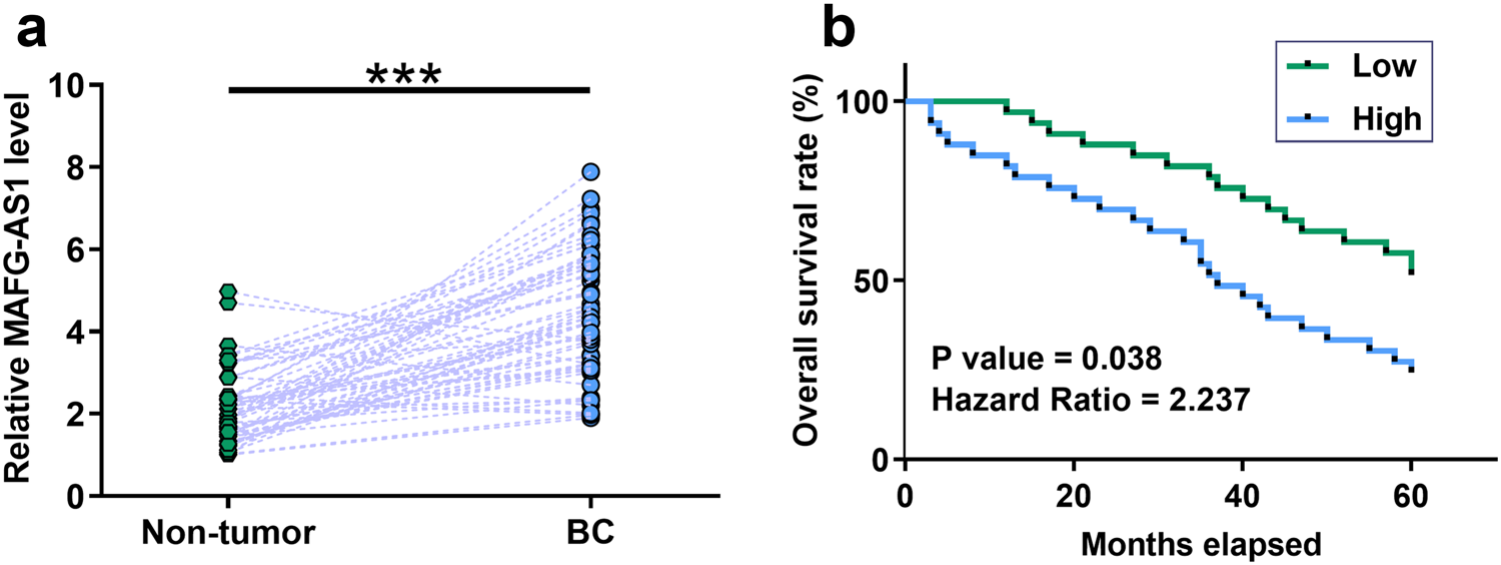
MAFG-AS1 was upregulated in BC and predicted poor survival. The expression levels of MAFG-AS1 in both BC and non-tumor tissues of 66 BC patients were measured by qPCR. Experiments were repeated 3 times and mean values were presented (**a**). ***, *p* < 0.001. Survival curves were plotted for high and low MAFG-AS1 level groups (*n* = 32, median level of MAFG-AS1 in BC tissues as cutoff value) based on the 5-year follow-up data. Survival curves were compared by log-rank test (**b**)

TCGA and GTEx dataset was explored using an online program GEPIA (http://gepia.cancer-pku.cn/) and it was observed that the expression levels of MAFG-AS1 were obviously higher in BC tissues than that in non-tumor tissues (2.05 vs. 0.65). The expression levels of MAFG-AS1 in both BC and non-tumor tissues of the 66 BC patients were measured by qPCR to further confirm its upregulation. Compared to non-tumor tissues, the expression levels of MAFG-AS1 were significantly higher in BC tissues (Fig. 2a, *p* < 0.001). Comparison of survival curves of high and low MAFG-AS1 level groups showed that patients in the high MAFG-AS1 level group experienced significantly lower overall survival rate (Fig. 2b, *p* = 0.038, Hazard Ratio = 2.237). Chi-squared test showed that the expression levels of MAFG-AS1 were not correlated with patients’ age, gender, clinical stage, tumor diameter, multiplicity, smoking, and drinking habits (Table 1). Therefore, MAFG-AS1 is likely an independent prognostic factor for BC.

**Table 1:** Correlations between expression levels of MAFG-AS1 and patients’ clinical data

Items	Groups	Cases	High-expression	Low-expression	χ^2^	*p* value
Gender	Male	42	20	22	0.26	0.61
	Female	24	13	11		
Age (years)	> 55	32	14	18	0.97	0.32
	≤ 55	34	19	15		
Clinical Stage	I	5	2	3	1.05	0.79
	II	15	8	7		
	III	23	10	13		
	IV	23	13	10		
Tumor diameter (cm)	> = 3	36	17	19	0.24	0.62
	< 3	30	16	14		
Multiplicity	Single	45	20	25	1.74	0.19
	Multiple	21	13	8		
Smoking	Yes	36	17	19	0.13	0.72
	No	30	16	15		
Drinking	Yes	44	20	24	1.09	0.30
	No	22	13	9		

### MAFG-AS1 was not correlated with miR-125b-5p but was correlated with SphK1

**Fig. 3:**
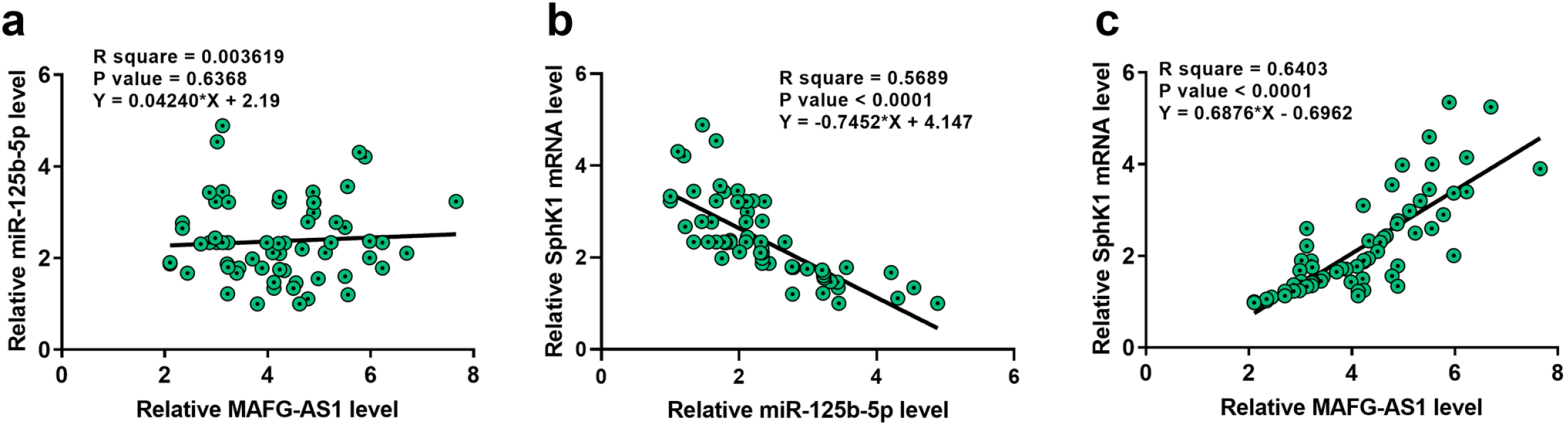
MAFG-AS1 was not correlated with miR-125b-5p but was correlated with SphK1. Correlations between MAFG-AS1 and miR-125b-5p (**a**), between miR-125b-5p and SphK1 (**b**), and between MAFG-AS1 and SphK1 (**c**) were analyzed by Pearson’s correlation coefficient

Correlation between the expression levels of MAFG-AS1 and miR-125b-5p across BC tissues was analyzed by Pearson’s correlation coefficient. It showed that MAFG-AS1 and miR-125b-5p were not correlated across BC tissues (Fig. 3a). SphK1 is a downstream target of miR-125b-5p [11], and it was shown to be involved in the development of BC [10]. Here, we also analyzed the correlations between miR-125b-5p and SphK1, as well as between MAFG-AS1 and SphK1 across BC tissues. It showed that the expression levels of SphK1 and miR-125b were negatively correlated (Fig. 3b), while the expression levels of SphK1 and MAFG-AS1 were positively correlated (Fig. 3c).

### MAFG-AS1 and miR-125b-5p did not affect the expression of each other in HT-1197 and HT-1376 cells

**Fig. 4:**
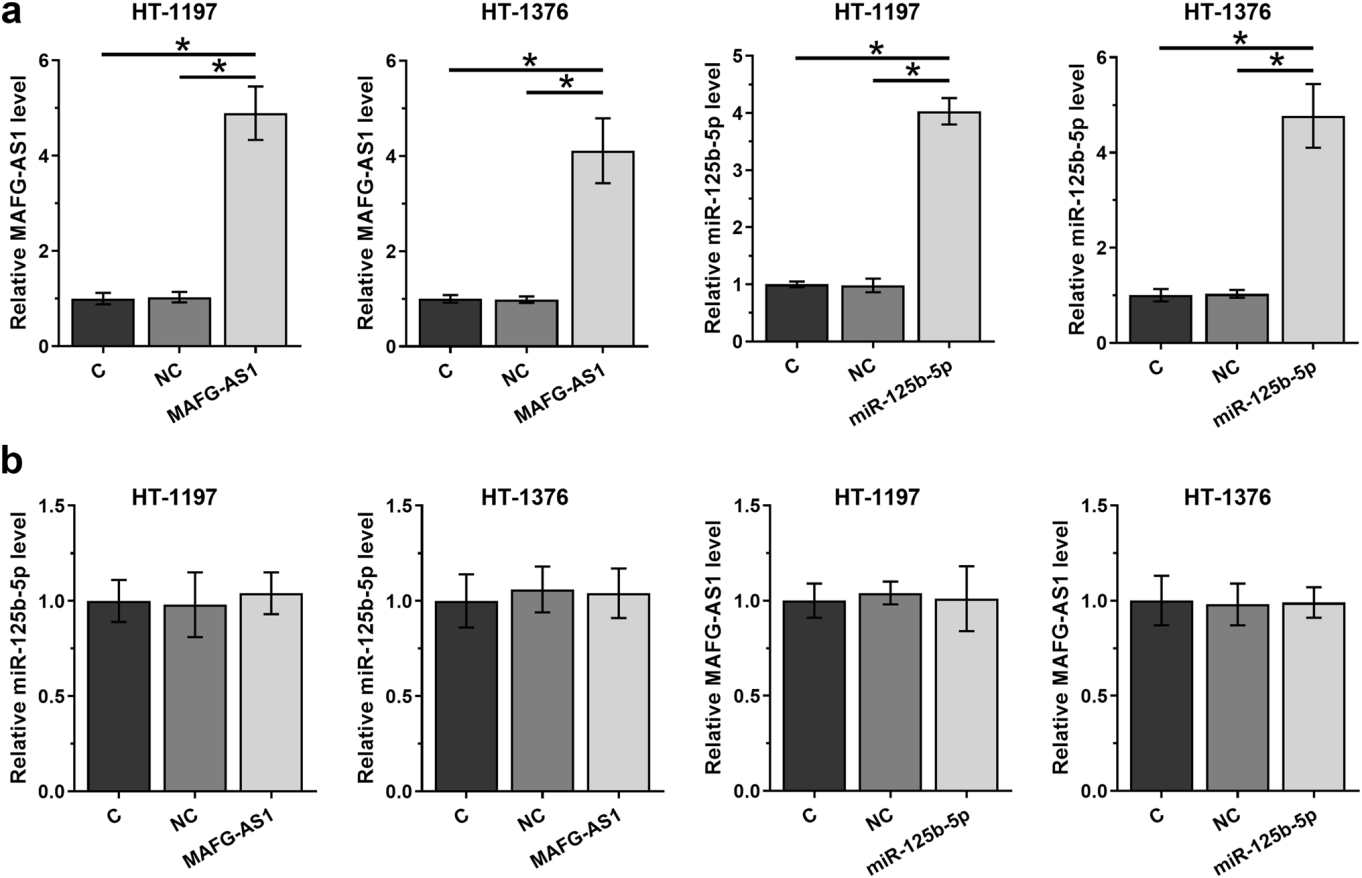
MAFG-AS1 and miR-125b-5p did not affect the expression of each other. HT-1197 and HT-1376 cells were transfected with MAFG-AS1 expression vector or miR-125b-5p mimic to analyze the interaction between them (**a**). The effects of overexpression of MAFG-AS1 and miR-125b-5p on the expression of each other were also analyzed by qPCR (**b**). RT-qPCR was performed at 48 h post-transfection. All experiments were repeated 3 times and mean values were presented. *, *p* < 0.05

HT-1197 and HT-1376 cells were transfected with MAFG-AS1 expression vector or miR-125b-5p mimic to analyze the interaction between them. Overexpression of MAFG-AS1 and miR-125b-5p were confirmed by qPCR at 48 h post-transfection (Fig. 4a, *p* < 0.05). Compared to C and NC groups, overexpression of MAFG-AS1 did not affect the expression of miR-125b-5p, and overexpression of miR-125b-5p also did not alter the expression of MAFG-AS1 (Fig. 4b, *p* < 0.05). Therefore, MAFG-AS1 is unlikely a target of miR-125b-5p.

### Overexpression of MAFG-AS1 resulted in upregulation of SphK1 in HT-1197 and HT-1376 cells

**Fig. 5:**
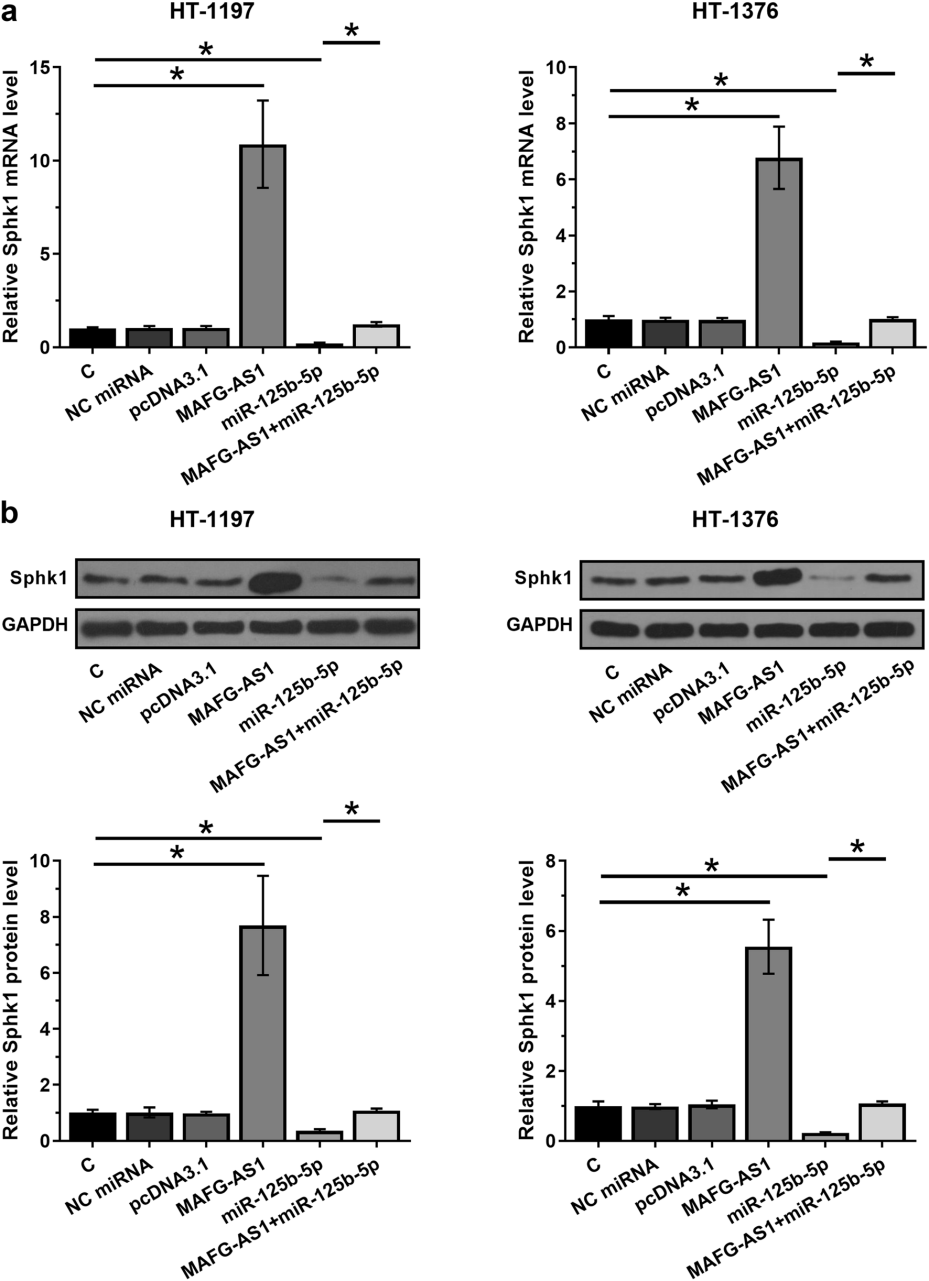
Overexpression of MAFG-AS1 led to the upregulated SphK1. The effects of MAFG-AS1 and miR-125b-5p on the expression of SphK1, a direct target of miR-125b-5p, were analyzed by qPCR and western blot at mRNA (**a**) and protein (**b**) levels, respectively. RT-qPCR and Western blot were performed at 48 h post-transfection. All experiments were repeated 3 times and mean values were presented. *, *p* < 0.05

Data presented above suggested that MAFG-AS1 is an endogenous sponge of miR-125b-5p. To test this possibility, the effects of MAFG-AS1 and miR-125b-5p on the expression of SphK1, a direct target of miR-125b-5p, were analyzed by qPCR and western blot at mRNA (Fig. 5a) and protein (Fig. 5b) levels, respectively. Compared to C group, downregulation of SphK1 was observed after the overexpression of miR-125b-5p (*p* < 0.05). Overexpression of MAFG-AS1 resulted in the upregulation of SphK1 and inhibited the effect of overexpression of miR-125b-5p (*p* < 0.05). It is worth noting that SphK1 was upregulated in BC tissues in comparison to that in corresponding non-tumor tissues by analyzing the TCGA and GTEx dataset (12.03 vs. 6.36).

### MAFG-AS1 promoted BC cell proliferation, migration, and invasion by regulating the miR-125b-5p/SphK1 axis

**Fig. 6:**
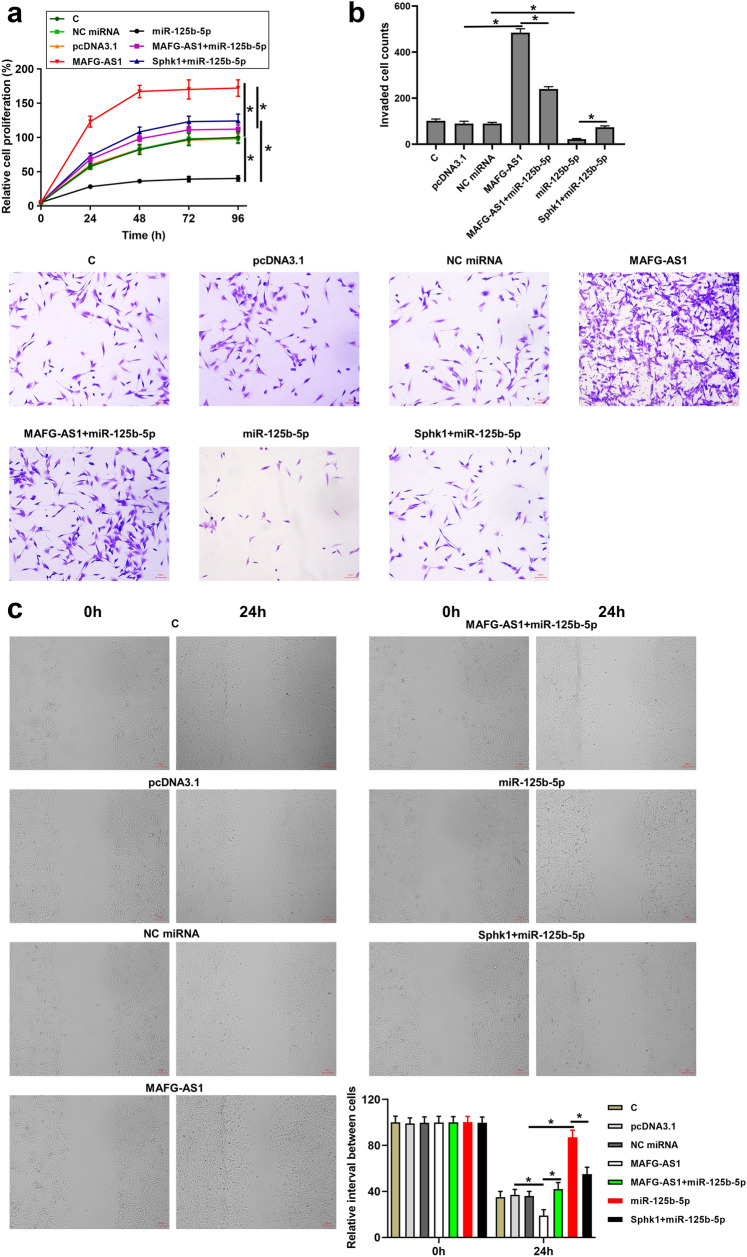
MAFG-AS1 promoted BC cell proliferation, migration, and invasion by regulating miR-125b-5p/SphK1 axis. HT-1197 cells were transfected with MAFG-AS1 expression vector, SphK1 expression vector or miR-125b-5p mimic. Cell proliferation was detected by CCK-8 assay (**a**). Cell invasion was detected by Transwell invasion assay (**b**). Cell migration was detected by wound healing assay (**c**). All experiments were repeated 3 times and mean values were presented. *, *p* < 0.05

To investigate the function of MAFG-AS1 in BC cells, HT-1197 cells were transfected with MAFG-AS1 expression vector. We found that overexpression of MAFG-AS1 promoted BC cell proliferation (Fig. 6a, *p* < 0.05), invasion (Fig. 6b, *p* < 0.05), and migration (Fig. 6c, *p* < 0.05). However, the effects of overexpression of MAFG-AS1 were alleviated by the overexpression of miR-125b-5p (Fig. 6, *p* < 0.05). In addition, HT-1197 cells were also transfected with miR-125b-5p mimic. It showed that overexpression of miR-125b-5p obviously inhibited BC cell proliferation (Fig. 6a), *p* < 0.05), invasion (Fig. 6b, *p* < 0.05), and migration (Fig. 6c), *p* < 0.05). However, the effects of overexpression of miR-125b-5p were attenuated by the overexpression of SphK1 (Fig. 6, *p* < 0.05). Therefore, MAFG-AS1 may has an oncogenic role in BC by regulating the miR-125b-5p/SphK1 axis.

## Discussion

The involvement of MAFG-AS1 in BC was investigated in this study. We found that MAFG-AS1 level was markedly upregulated in BC tissues. Functional analysis revealed that MAFG-AS1 promoted BC cell proliferation, migration, and invasion by regulating the miR-125b-5p/SphK1 axis. MAFG-AS1 has been characterized as an oncogenic lncRNA in several types of cancer. In colorectal cancer, MAFG-AS1 is overexpressed and may sponge miR-147b to upregulate oncogenic NDUFA4, thereby promoting cancer development and progression [12]. In hepatocellular carcinoma, MAFG‑AS1 is also overexpressed and downregulates miR‑6852 to promote the proliferation, migration, and invasion of cancer cells [13]. Our study is the first to report the overexpression of MAFG‑AS1 in BC. In addition, we also observed that overexpression of MAFG-AS1 promoted BC cell proliferation, migration, and invasion. Therefore, MAFG-AS1 is an oncogenic lncRNA in BC.

In this study we performed a 5-year follow-up study on the 66 patients. During the follow-up, 40 patients died of BC, accounting for 60.6% of all BC patients. The high mortality rate observed in this study is likely due to the fact that most patients in this study were diagnosed at stage III or IV. Survival rate of BC is generally poor [15, 16]. We also showed that high expression levels of MAFG‑AS1 in BC might predict the poor survival. Therefore, measuring the expression levels of MAFG‑AS1 in BC tissues before therapy may assist the prognosis of BC, thereby guiding the determination of therapeutic approaches and improving patient’s survival. However, the accuracy of using MAFG‑AS1 as a prognostic factor for BC remains to be further elucidated.

The interaction between MAFG-AS1 and miR-125b-5p has been predicted by IntaRNA software. Here, we confirmed their interaction by performing dual luciferase activity assay. However, we found that the expression levels of MAFG‑AS1 and miR-125b-5p in BC tissues were not obviously correlated. In addition, MAFG‑AS1 and miR-125b-5p didn’t regulate the expression of each other. Therefore, MAFG‑AS1 is unlikely a target of miR-125b-5p. SphK1, a downstream target of miR-125b-5p, has been found to promote BC cell proliferation and migration [10, 11]. In the present study, we found that SphK1 was negatively correlated with miR-125b-5p, while it was positively correlated with MAFG-AS1 across BC tissues. Moreover, overexpression of MAFG‑AS1 upregulated the expression of SphK1 in BC cells, and attenuated the inhibitory effects of miR-125b-5p on the expression of SphK1. Taken together, our data suggested that MAFG‑AS1 may sponge miR-125b-5p to upregulate SphK1.A previous study found that miR-125b-5p can inhibit the proliferation and migration of BC cells by targeting SphK1 [11]. Consistently, we also found that overexpression of miR-125b-5p inhibited the proliferation, migration, and invasion of BC cells, while its effects were attenuated by overexpression of SphK1. Moreover, we revealed that overexpression of MAFG-AS1 promoted BC cell proliferation, migration, and invasion, while its effects were alleviated by overexpression of miR-125b-5p. Collectively, MAFG-AS1 may exert its oncogenic role by regulating the miR-125b-5p/SphK1 axis in BC.

Although previous studies and our study both demonstrated the oncogenic role of SphK1 in BC [10, 11], the detailed mechanism by which SphK1 regulated the tumorigenesis of BC needs to be further elucidated. A previous study has found that the SphK1 inhibitor SKI II can suppress the activation of Wnt/β-catenin pathway [17]. Therefore, we speculated that SphK1 may play the oncogenic role in BC by regulating the Wnt/β-catenin pathway. Further studies are needed to confirm our speculation.

Our study has some limitations. Firstly, the sample size of BC patients is not big enough. Our future study will add more samples to further confirm our findings. Moreover, although we have verified that MAFG‑AS1 can bind with miR-125b-5p by performing dual luciferase activity assay, whether the binding happens in physiological condition is unclear given the low expression of lncRNAs and miRNAs in the natural state. Our further study will perform RNA pulldown assay and RIP assay to further clarify the interaction between MAFG‑AS1 and miR-125b-5p in physiological condition.

## Conclusions

In conclusion, our study is the first to report the overexpression of MAFG-AS1 in BC. We also showed that MAFG-AS1 could upregulate SphK1 by sponging miR-125b-5p to promote the proliferation of BC cells. We, therefore, proposed a novel MAFG-AS1/miR-125b-5p/SphK1 pathway in BC. Future studies may focus on the clinical applications of MAFG-AS1/miR-125b-5p/SphK1 pathway in the treatment of BC.

## Data Availability

The analyzed data sets generated during the study are available from the corresponding author on reasonable request.

## Abbreviations

BC: Bladder cancer
S1P: Sphingosine-1-phosphate
lncRNA: Long non-coding RNA
